# Dermoscopy as a Useful Tool in Differentiation of Genital Lesions: Lichen Sclerosus

**DOI:** 10.5826/dpc.1104a133

**Published:** 2021-10-01

**Authors:** Ružica Jurakić Tončić, Tatjana Matijević, Marija Milković Periša, Daška Štulhofer Buzina, Romana Čeović

**Affiliations:** 1 Department of Dermatology and Venereology, School of Medicine, University of Zagreb, University Hospital Centre Zagreb, Zagreb, Croatia; 2 Department of Dermatology and Venereology, University Hospital Centre Osijek, Osijek, Croatia; 3 Department of Pathology, School of Medicine, University of Zagreb and Department of Pathology and Cytology, University Hospital Centre Zagreb, Zagreb, Croatia

**Keywords:** genital lesion dermoscopy, lichen sclerosus, venerology

## Introduction

Lichen sclerosus (LS) is a chronic inflammatory disease of unknown etiology that most commonly affects the anogenital area, with a higher prevalence in women. It is characterized by ivory-white sclerotic plaques with thin wrinkled skin surface and frequently seen erosions and fissures [1]. Pruritus is the most common symptom of genital lichen sclerosus (GLS), significantly impairing the quality of life and can be accompanied by dyspareunia in women. Scarring can lead to loss of normal anatomical architecture along with disease progression [1]. Early diagnosis and proper treatment are of particular importance. Although in most cases of advanced disease diagnosis is usually straightforward, early diagnosis can sometimes be challenging and present some diagnostic uncertainties.

## Case Presentation

**Figure 1:**
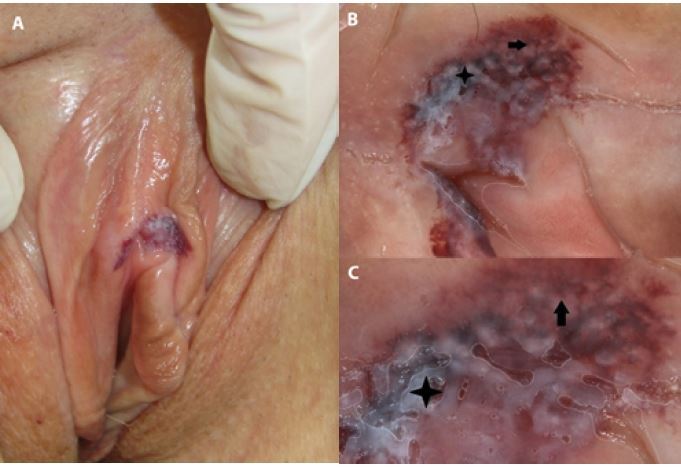
Clinical and dermoscopic presentation of the lesion at the first visit. (A) Clinical presentation. (B, C) Dermoscopic presentation of the lesion showing multiple red and white structureless areas (arrow) with a blue-white zone on the left part of the lesion (asterisk).

**Figure 2:**
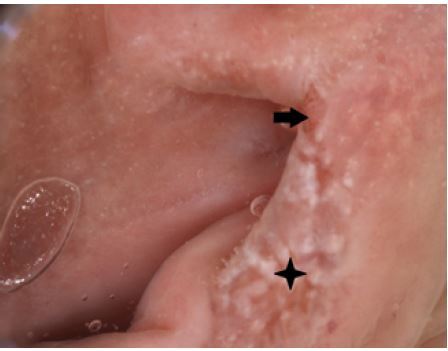
Dermoscopic presentation of the lesion at the second visit: multiple patchy structureless white and milky-pinkish areas on a whitish background (asterisk) with a small erosion area (arrow).

A 59-year-old, otherwise healthy woman, presented with a dark macule on her vulva which appeared spontaneously a few weeks before her visit. The patient had a fair phototype, multiple solar lentigines, and other signs of sun-damaged skin. Clinical examination revealed a notably dark asymmetrical pigmented lesion with indistinct borders on the clitoral hood (Figure 1 A). Dermoscopy showed multiple red and white structureless areas with a blue-white zone on the left part of the lesion (Figure 1, B and C). The observed dermoscopic features with a chaotic arrangement was alerting, biopsy was recommended also considering the fact that this was a newly-onset lesion. Due to its hemorrhagic component, the lesion was first suspected to be a vascular tumor, and the main clinical concern was to exclude malignancy, especially melanoma. However, the biopsy was not performed immediately due to coronavirus pandemic and health system reorganization. A month after the first visit and before she underwent the biopsy, the patient came again to our clinic to report the change of the lesion. Another clinical examination of the suspected lesion showed that hemorrhagic component completely disappeared and now only a delicate area of leukoplakia was noticed. In addition, a similar finding was also detected on the right labium minus. Control dermoscopy was performed. Multiple patchy structureless white and milky-pinkish areas on a whitish background with a small erosion area were observed (Figure 2). The patient also complained about pruritus affecting the genital area. Biopsy was recommended again, this time not because of a suspected malignant lesion, but because of LS suspicion. Full-thickness skin biopsy was performed, and the histological examination showed hyalinization of the dermis with moderate inflammatory infiltrate, indicating LS. After ruling out malignancy and the confirming LS diagnosis, a potent local corticosteroid was prescribed with a good control of the disease and the regression of all clinical signs and symptoms.

## Conclusion

Genital LS can present a diagnostic challenge in its early stages. It is known that LS plaques can commonly show signs of hemorrhage due dermal capillaries’ fragility and chronic scratching, but dermal hemorrhage is quite rare as a first sign of the disease [2]. Also, it is notable from our case that purpura in set of genital LS lesion can give bizarre dermoscopic appearance, mimicking a potentially malignant lesion.

Dermoscopic patterns of large case series of genital LS were observed in few studies to date, and the features that we observed on the second visit were mentioned as one of the most consistent findings [2]. However, even though dermoscopy can help establishing a diagnosis of LS, its role is still limited. To confirm diagnosis histopathological examination can sometimes be required in cases of diagnostic uncertainty, especially when the disease is in its early stages, and to rule out malignant diagnoses affecting the genital region. We believe that genital LS can be considered as a possible differential diagnosis of melanoma in the genital region.
